# Maine Veterinary Medical Association

**Published:** 1901-02

**Authors:** F. E. Freeman

**Affiliations:** Secretary


### MAINE VETERINARY MEDICAL ASSOCIATION.

THE annual meeting was held at Lewiston, January 9th, at 7 P.M., President Dr. A. Joly in the Chair. The members and veterinarians present were Drs. Joly, Russell, West, Salley, Blakely, Goddard, Fairbanks, Inglas, Goff, Brackett, and Freeman.

The minutes of the last meeting were approved as read.

President Joly, in his interesting annual address, gave some very good advice to the members, and urged that a clinic be held at each meeting.

The subject of a veterinary bill to be presented during the present Legislature was discussed, and Drs. Russell, Joly, and Blakely were made a committee to present the same.

Dr. W. E. Fairbanks, of Lewiston, and Dr. J. H. Goddard, of Lewiston, were elected members of the Association.

The following officers were elected for the ensuing year: President, A. Joly, Waterville; Vice-President, C. L. Blakely, Augusta; Secretary, F. E. Freeman, Rockland; Treasurer, I. L. Salley, Skowhegan. Censors: F. L. Russell, Orono; W. L. West, Belfast; J. H. Goddard, Lewiston.

Dr. W. L. West read a very able paper on "Surgical Dentistry," which was well discussed. The next meeting will be held at Waterville in April. There will be a clinic at Dr. Joly's hospital.

F. E. FREEMAN, V.S.,
Secretary.

